# Outcome of different reconstruction options using allografts in revision total hip arthroplasty for severe acetabular bone loss: a systematic review and meta-analysis

**DOI:** 10.1007/s00402-023-04843-9

**Published:** 2023-03-27

**Authors:** André Strahl, Christoph Kolja Boese, Christian Ries, Jan Hubert, Frank Timo Beil, Tim Rolvien

**Affiliations:** Division of Orthopaedics, Department of Trauma and Orthopaedic Surgery, University Medical Center Hamburg-Eppendorf, Martinistr. 52, 20246 Hamburg, Germany

**Keywords:** Allograft, Revision, Acetabular bone loss, Paprosky classification, Meta-analysis

## Abstract

**Introduction:**

Several studies have reported good to excellent outcomes of revision total hip arthroplasty (rTHA) using allografts for treating severe acetabular bone defects. However, precise information on the impact of allograft type and reconstruction method is not available.

**Material and methods:**

Systematic literature search was performed in Medline and Web of Science including patients with acetabular bone loss classified according to the Paprosky classification who underwent rTHA involving the use of allografts. Studies with a minimum follow-up of 2 years published between 1990 and 2021 were included. Kendall correlation was applied to determine the relationship between Paprosky grade and allograft type use. Proportion meta-analyses with 95% confidence interval (CI) were performed to summarize the success of various reconstruction options, including allograft type, fixation method, and reconstruction system.

**Results:**

Twenty-seven studies met the inclusion criteria encompassing 1561 cases from 1491 patients with an average age of 64 years (range 22–95). The average follow-up period was 7.9 years (range 2–22). Structural bulk and morselized grafts were used in equal proportions for all Paprosky acetabular defect types. Their use increased significantly with the type of acetabular defect (*r* = 0.69, *p* = 0.049). The overall success rate ranged from 61.3 to 98.3% with a random effect pooled estimate of 90% [95% CI 87–93]. Trabecular metal augments (93% [76–98]) and shells (97% [84–99]) provided the highest success rates. However, no significant differences between reconstruction systems, allograft types and fixation methods were observed (*p* > 0.05 for all comparisons).

**Conclusion:**

Our findings highlight the use of bulk or morselized allograft for massive bone loss independent of Paprosky classification type and indicate similar good mid- to long-term outcomes of the different acetabular reconstruction options using allografts.

**Clinical trial registration:**

PROSPERO: CRD42020223093.

**Supplementary Information:**

The online version contains supplementary material available at 10.1007/s00402-023-04843-9.

## Introduction

Reconstruction of severe acetabular bone defects during revision total hip arthroplasty (rTHA) represents a challenging procedure with a variety of available options [1]. Current projections describe a significant increase in rTHA of 43–70% by 2030, especially in patient groups aged 55–64 and 65–74 years [2]. To ensure the best possible service life of the prosthesis, defects of the acetabular bone stock must be addressed using a suitable reconstruction method. This includes the selection of appropriate implants during surgery planning along with the precise classification of the bone defect [3]. The appropriate type of acetabular bone graft is typically selected depending on the classification of the bone stock deficit. While cavitary defects may be restored with cancellous morselized auto- or allografts, larger segmental bone stock defects are usually addressed with structural (or bulk) cortico-cancellous auto- or allografts [4].

First described in 1994, the Paprosky classification was developed to grade acetabular bone loss [5] and has become the most widely used acetabular defect classification system worldwide [6]. Besides the description of the severity of bone defects, the classification was also developed to predict the required bone graft and implants. Whereas Paprosky types 1 and 2 defects are mostly treated with morselized bone graft, a bulk graft is recommended for type 3 defects [5, 7].

Several studies have documented good to excellent clinical and radiological outcomes of rTHA using allografts [8–11]. Our group has previously demonstrated successful histological incorporation of morselized and bulk allografts in acetabular reconstruction, thereby providing a scientific basis for their successful use in rTHA [12, 13]. However, differences in outcomes according to type of allograft, bone defect and reconstruction devices have not been systematically investigated. Current reviews focused on overall treatment options but not on the reconstruction device and bone graft in relation to the defect type [1, 14, 15]. So far these reviews depicted that trabecular metal (TM) systems, comprising augments and shells, demonstrated the best results for large acetabular defect reconstruction, referring to re-revisions and radiographic loosening [1, 14].

Therefore, aim of the present study was to provide a systematic overview of mid- and long-term outcomes of rTHA using morselized or bulk allografts considering different surgical reconstruction options. Specifically, this systematic review and meta-analysis compiled studies with the purpose to (a) investigate the use of allograft types according to the acetabular bone loss classified according to Paprosky and (b) to evaluate the success rates of various cup or reconstruction devices combined with morselized or bulk allografts.

## Methods

The findings are described according to MOOSE guidelines for reporting meta-analyses [16]. Study protocol was registered with the international prospective register of systematic reviews (http://www.crd.york.ac.uk/PROSPERO/) and can be accessed under registration code CRD42020223093.

### Search strategy

Medline (National Library of Medicine) and Web of Science (Clarivate Analytics) were screened for literature published from 1st January 1990 to 31th December 2021. The search was supplemented by records identified through the bibliographic lists of included references. The detailed search history is listed in Suppl. Table 1.

Titles and abstracts were screened. If title or abstract did not provide sufficient information, full texts were accessed. Two investigators (AS, TR) independently screened titles, abstracts, and full texts according to predefined inclusion and exclusion criteria (Table 1). Incongruities were resolved by consensus discussion. To minimize screening bias, the systematic review tool Rayyan was used [17].

**Table 1:** Inclusion and exclusion criteria

*Inclusion criteria*
Randomized controlled trials
Prospective cohort studies
Retrospective follow-up studies
Case series
Revision total hip arthroplasty with morselized/structural allografts
acetabular loss described with the classification of Paprosky
Sample size > 20 patients
Minimum follow-up > 2 years
Patients > 18 years of age
Written in English language
*Exclusion criteria*
Conference abstracts
Case reports
Patients with primary total hip replacement
Mixed use of allo-, auto- or heterografts
Oncological patients
Publication date before 1990

### Quality assessment

Two investigators (AS, CKB) independently evaluated the quality of the full texts with consensus discussion in case of discrepancies. Quality assessment was performed with the modified Downs and Black checklist [18] classifying quality according to four quality levels: excellent (28–26 points), good (25–20 points), fair (19–15 points), and poor (≤ 14 points) [19].

### Data analysis and meta-analysis

For data synthesis, the main findings are summarized in Table 2. Cage fractures, broken hooks/plates, broken or loose fixation screws, unspecified mechanic failures and unstable cups were summarized as combined implant failure. The infection rate is compounded from early post-operative and late (deep) infections, and the pooled dislocation rate integrates cases with early post-operative and recurrent dislocation. The main outcome “success rate” was evaluated for each included study by subtracting the number of implant failures, aseptic loosening, and infections from the total number of surgical procedures. Dislocation was not included in the success rate since confounding factors had a significant impact on the occurrence of dislocation [20]. The investigated surgical reconstruction devices comprise reconstruction cages, meshes, cemented and cementless cups, TM acetabular augment, TM shells and customized special cups. Studies that used different allografts or reinforcement devices were excluded from this analysis.

**Table 2:** Study characteristics and outcomes

Study	Revision period	No. hips /patients	Sex M:F	Mean age (± SD/range)	Mean no. previous surgeries (range)	Follow-up in years (± SD/range)	Acetabular defect	Type of allograft	Type of reinforcement hardware and cup	Cup fixation
*Morselized chip allograft*										
Akel et al. [33]	2000 to 2015	56/54	13:41	57 (29–79)	–	7.1 (± 3.7)	IIA 2 IIB 10 IIC 12 IIIA 11 IIIB 21	Morselized	Titanium reconstruction cage with flanges (contour TM) Polyethylene cup	Cemented
Babis et al. [44]	2003 to 2007	62/62	17:45	62.4 (37–81)	1.6 (1–3)	5.0 (3–7.8)	IIIA 62	Morselized	Oblong implant with sideplates and hook (procotyl E) Polyethylene cup	Cementless
Borland et al. [25]	2004 to 2008	24/24	13:11	62 (24–87)	1 to 4	4.9 (2.7–6.8)	IIIA 15 IIIB 9	Morselized	Wedge type trabecular metal augment Low-profile exeter cup	Cemented
Dennis [27]	–	24/24	7:17	67.8 (44–82)	1–4	4.0 (2–6.5)	IIIB 24	Morselized	Costume triflanged acetabular component Polyethylene cup	Cementless
Ding et al. [34]	2005 to 2010	29/28	–	61 (43–75)	1.4 (1–3)	6.1 (4–9)	IIB 3 IIC 2 IIIA 14 IIIB 10	Morselized	Revision cup system with acetabular hook and flanges Hemispherical titanium alloy cup	Cemented
El-Kawy et al. [49]	1994 to 1998	28/27	10:17	72.8 (52–87)	1.3 (1–4)	6.0 (4.0–7.6)	IIIA 23 IIIB 5	Morselized	I.D. polyethylene flanged OGEE socket Polyethylene cup	Cemented
Gilbody et al. [40]	1995 to 2001	304/292	107:185	70.3 (34–95)	1.3 (1–4)	12.4 (10–16)	I 7 IIA 82 IIB 93 IIC 49 IIIA 49 IIIB 24	Morselized	Meshes (X-change) Acetabular components: exeter concentric implants, OGEE, exceter Contemporary, McKee-Arden, Muller	Cemented
Holt et al. [24]	–	26/26	8:18	69.2 (44–82)	1 to 4	4.5 (2–7.1)	IIIB 26	Morselized	Costume triflanged acetabular component Polyethylene cup	Cementless
Hosny et al. [48]	2009 to 2013	26/25	11:14	71 (49–91)	1.8 (1–4)	4.1 (2.5–6.5)	IIB 2 IIC 4 IIIA 12 IIIB 8	Morselized	Titanium reconstruction ring (GAP II) Contemporary hooded Exeter cups	Cemented
Lee et al. [47]	1992 to 2000	71/62	–	–	–	12 (10–14.7)	I 13 IIA 14 IIB 17 IIC 20 IIIA 4 IIIB 3	Morselized	No cage/ring 42 trilogy components, 29 Harris–Galante II components	Cementless
Philippe et al. [32]	1987 to 1995	95/95	29:66	69.5 (42–86)	–	8 (5–13)	IIA/B 12 IIIA/B 83	Morselized	Reinforcement rings: - Eichler - Ganz - Müller - Burch–Schneider Polyethylene cup	Cemented
Quarto et al. [37]	1998 to 2010	40/40	8:32	71.4 (33–93)	0 to 2	14.3 (10–22)	IIIA 27 IIIB 13	Morselized	Burch–Schneider cage Polyethylene cup	Cemented
Sancho Navarro et al. [41]	1995 to 2005	57/45	20:25	71 (48–84)	–	10.1 (4.5–14.3)	IIIA 57	Morselized	Hemispheric Furlong hydroxyapartite-coated cups	Cementless
Siegmeth et al. [38]	2002 to 2005	34/34	15:19	64 (37–97)	1.4 (0–6)	2.8 (2–4.6)	IIA 4 IIIB 2 IIIB 1 IIIA 19 IIIB 8	Morselized	Modular tantalum augment with trabecular metal acetabular Shells and cortical screws Polyethylene cup	Cemented
Torres-Campos et al. [42]	1999 to 2012	67/64	25:39	71.1 (46–88)	–	5.1 (2.2–12)	I 3 IIB 9 IIC 19 IIIA 18 IIIB 2	Morselized	Burch–Schneider cage 60 “allpoli” Muller cups 5 Trident-type constrained ring 2 exeter-typer cups	Cemented
van Egmond et al. [43]	1993 to 2003	27/25	5:20	63 (42–82)	–	8.8 (3–14.1)	IIB 4 IIIA 14 IIIB 9	Morselized	Metallic meshes polyethylene cup - 11 Muller cups - 8 exeter cups - 8 Charnley cups	Cemented
Xiao et al. [30]	2007 to 2016	28/28	13:15	56.4 (36–75)	1.3 (1–4)	6.6 (3.2–11.8)	IIIA 13 IIIB 15	Morselized	Reconstruction cage polyethylene cup	Cemented
*Structural bulk allograft*										
Chang et al. [26]	–	20/20	10:10	56.2 (43–68)	–	5.4 (3.3–10.3)	IIIA/B 20	Bulk	Tantalum trabecular metal Acetabular cup	Cementless
Gibon et al. [39]	1995 to 2016	37/36	9:27	65.1 (37–84.2)	1.5 (1–5)	8.2 (5–19.3)	IIIA 14 IIIB 23	Bulk	Kerboull acetabular reinforcement device Polyethylene cup till 2006, remelted HXLPE cup components after 2006	Cemented
Hsu et al. [46]	2003 to 2010	31/29	11:18	59 (37–79)	2.0 (1–4)	5.5 (3–10.5)	IIIA 17 IIIB 14	Bulk	Burch–Schneider cage Polyethylene cup	Cemented
Makita et al. [50]	2000 to 2005	65/59	10:49	59.1 (23–85)	–	11.2 (2–14)	IIIA 47 IIIB 18	Bulk	Kerboull acetabular reinforcement device polyethylene cup	Cemented
Peng et al. [31]	1989 to 2002	104/96	54:42	60 (31–84)	–	14.5 (12–22)	IIA/B 25 IIIA/B 79	Bulk	No cage/ring 80 trilogy components, 24 Harris–Galante II components	Cementless
Piriou et al. [28]	1985 to 1996	20/17	12:5	56 (27–75)	3.0 (1–10)	5 (4–10)	IIIB 20	Bulk	No cage/ring Acetabular component with no further Information	Cemented
Piriou et al. [29]	1984 to 1995	140/135	–	61 (22–87)	2.0 (1–10)	10 (5–16)	I 8 IIA 27 IIB 61 IIC 16 IIIA 26 IIIB 2	Bulk	No cage/ring acetabular component with no further Information	Cemented
Prieto et al. [35]	2000 to 2010	58/56	10:46	64 (35–86)	–	5.4 (2–12)	IIA 6 IIB 12 IIC 12 IIIA 11 IIIB 17	Bulk	Highly porous trabecular metal shell hemispherical cup	Cementless
Regis et al. [45]	1992 to 2000	65/65	16/49	60 (29–83)	–	14.6 (10–18.9)	IIIA 27 IIIB 38	Bulk	Burch–Schneider cage polyethylene cup	Cemented
Sporer et al. [36]	1985 to 1990	23/23	7:16	61 (37–77)	3.2 (1–4)	10.3 (7–15)	IIIA 23	Bulk	Porous coated hemispherical shell	Cementless

Study	Clinical outcome									Radiographic appearances		
	HHS (SD/range)	MdA (SD/range)	No. of revisions	Time to revision in years (SD/range)	No. of implant failure	No. of aseptic loosening	No. of dislo-cation	No. of infection	Other	Radiolucency	Other	Cup survival (95% CI)
*Morselized chip allograft*												
Akel et al. [33]	45.9 (± 7.0)	–	5 revisions - cage fracture - cage migration - aseptic cage loosening - infection - migration/lateralised center of rotation	2.6 (± 2.2)	1 cage fracture (5th year)	1 cage loosening (1st year)	–	1 infection (1st year)	–	1 stable radiolucency	2 cage migration (1st, 5th year) 1 ischium fractur (2nd year)	90.6% 5th year 91.5% latest FU
Babis et al. [44]	88.0 (40–98)	–	18 revisions 4 awaiting revision	2.7 (2.2–7.0)	1 broken hook/side plate	19 aseptic loosening	–	2 deep infection	1 femoral nerve palsy	22 radiolucent lines 12 minor radiolucent line < 1 mm	19 migration	98% (CI 96.2–99.9) 2nd year 71% (CI 68.7–71.3) 3rd year 64.5% (CI 62.5–68.8) 5th year
Borland et al. [25]	–	–	1 revision - augment fracture	1.1	1 fractured augment	–	0	0	–	2 progressive radiolucent zones 3 non-progres-sive lucency	5 migration > 5 mm	–
Dennis [27]	79 (68–89)	–	1 revision - aseptic component loosening	–	–	3 aseptic loosening (at 1,5th year)	2 post-operative dislocation	0	2 suspected superior gluteal nerve injuries	–	3 migration with screw disengage-ment	–
Ding et al. [34]	80 (71–98)	–	0 revisions	–	–	–	0	–	1 intra-muscula hemtoma 1 femoral/peroneal nerve palsy	–	1 migration > 5 mm	–
El-Kawy et al. [49]	78 (15–100)	–	1 revision - aseptic loosening/ cup migration	< 1 year	–	1 aseptic loosening in IIIB (within 1st year)	1 post-operative dislocation (few weeks after revision)	0	–	4 radiolucent lines < 2 mm	2 migration > 5 mm in IIIA and IIIB (1st, 5th year)	96.4% 6th year 92.85% 6th year aseptic loose
Gilbody et al. [40]	HHS_pain_ I 44.0 IIA 36.3 IIB 34.5 IIC 38.8 IIIA 40.0 IIIB 40.3 HHS_Function_ I 21.5 IIA 29.0 IIB 25.8 IIC 27.6 IIIA 28.2 IIIB 26.7	Pain IIA 5.1 IIB 5.0 IIC 5.5 IIIA 5.6 IIIB 5.0 Function IIA 3.5 IIB 3.2 IIC 3.3 IIIA 3.4 IIIB 3.0 ROM IIA 5.0 IIB 4.7 IIC 4.8 IIIA 5.3 IIIB 4.8	37 revisions - aseptic cup loosening - recurrent dislocation - infection	6.4 (0.2–14.7) (aseptic) 6.0 (1.9–13.1) (dislocation) 10.1 (infection)	–	33 aseptic loosening Including: IIA: 6 IIB: 5 IIC: 3 IIIA: 9 IIIB: 4	10 recurrent dislocation	1 deep infection (10th year)	1 pulmonary embolism 1 cerebro-vascualar accident 1 myocardial infarct 2 deep vein thrombosis 1 femoral nerve palsy 2 sciatic nerve palsy 3 trochanter fractures 1 calcal fracture 4 femoral shaft fractures 4 femoral perforations 1 pelvis penetration	–	Acetabular components:- 77 stable - 20 probably stable - 13 probably unstable - 15 definitely unstable: IIA 1 unstable IIB 8 unstable IIC 3 unstable IIIA 2 unstable IIIB 1 unstable	82.8% (CI 76.9–88.7) 13.5th year 85.9% (CI 81.0–90.8) 13.5th year aseptic loose
Holt et al. [24]	78.0 (68–89)	–	3 revisions - aseptic component loosening	about 1.5 years	3 ischial screw loosening	0	2 post-operative dislocation	0	2 suspected superior gluteal nerve injuries	–	3 migration with screw disengage-ment	–
Hosny et al. [48]	–	–	0 revisions	–	1 implant breakage at plate cup junction	–	1 post-operative dislocation (6th week)	0	2 femoral perforations	–	–	100% 4.1th year
Lee et al. [47]	92 (–)	–	3 revisions - aseptic loosening - fixation loss	4.0 (± 2.8)	1 fixation loss in IIIB (6th weeks)	2 aseptic loosening (both 6th year)	2 post-operative dislocation (< 6 week)	0	0	12 radiolucent lines < 2 mm	2 migration > 4 mm	95% 12th year
Philippe et al. [32]	71.1 (40–94)	14.8 (8–18)	11 revisions - aseptic loosening - dislocation - infection - migration	–	–	2 aseptic loosening	1 post-operative dislocation 7 recurrent dislocation	1 post-operative superficial infection 3 deep infection (< 2 years)	20 infections urinary tract 9 deep vein thrombosis 2 acute pulmonary oedema 2 pneumonia 2 femoral loosening	11 radiolucent lines < 1 mm 1 radiolucent lines < 1 mm; covering entire surface 2 progressive radiolucent lines > 1 mm	5 cage migration > 4 mm	77.9% (CI 62–93.4) 13th year revision and loosening
Quarto et al. [37]	78.1 (± 7.6)	–	2 revisions - infection - aseptic loosening	6.7	–	1 aseptic loosening	–	1 septic failure	–	12 progressive radiolucent lines	3 cage migration > 5 mm	95.0% mean FU 14.3 years
Sancho Navarro et al. [41]	–	17.2	2 revisions - aseptic loosening	3.5 (± 1.5)	–	2 aseptic loosening (2nd, 5th year)	–	1 post-operativewound infection 3 post-operative type IV infection	1 sciatic nerve palsy 1 intra-operative acetabular fracture 1 amaurosis for carotid thrombosis 1 abdominal distention	2 radiolucent lines < 2 mm 2 progressive radiolucent lines	–	–
Siegmeth et al. [38]	–	–	3 revisions - aseptic loosening - recurrent dislocation	1.3 (± 0.6)		2 aseptic loosening (1st year)	1 post-operative dislocation 1 recurrent dislocation (2nd year)	0	–	1 radiolucent line augment-bone	–	–
Torres-Campos et al. [42]	–	14.3 (7–18)	5 revisions - implant failure - dislocation - infection	–	2 mechanic implant failures 2 broken fixation screw	–	2 post-operative dislocation 2 recurrent dislocation	1 deep infection 3 post-operativewound infection	1 peri-prosthetic fracture 1 mobilisation of femoral rod	4 radiolucent lines	6 migration	93.4% 5th year 84.6% 10th year
van Egmond et al. [43]	71.6 (33–95)	–	3 revisions - aseptic loosening - unstable cup - infection	3.9 (± 2.0)	1 unstable cup	1 aseptic loosening	1 recurrent dislocation 2 dislocation hip	1 post-operative infection 1 deep infection	3 peri-prosthetic fracture 1 peri-operative stem perforation 1 peroneal nerve palsy	–	5 migration (1st, 1.9th, 5.8th, 8th, 8.1th year)	88% (CI 74.2–100) 10th year Revision 77.2% (CI 59–95.4) 10th year Loosening
Xiao et al. [30]	84.6 (55–94)	–	1 revision - aseptic cage loosening	5.1	1 broken fixation screw (5.1th year)	1 aseptic cage loosening (5.1th year)	1 recurrent dislocation (8th months)	0	1 sciatic nerve palsy 1 acute renal injury 2 femoral loosening (2nd year)	2 radiolucent lines < 2 mm	1 migration > 5 mm	–
*Structural bulk allograft*												
Chang et al. [26]	84.1 (77–91)	–	0 revisions	–	–	0	0	0	0	1 radiolucent line	1 migration > 3 mm	100% latest FU
Gibon et al. [39]	–	16.5 (10–18)	4 revisions - recurrent dislocation - infection - aseptic cup loosening - congenital hip disease with major bone graft resorption	8.6 (6–13.2)	0	1 aseptic acetabular component loosening	3 recurrent dislocation	0	1 femoral peri-prosthetic fracture 3 non-union of the great trochanter	–	8 migration > 5 mm 1 allograft screw migration	79.2% (CI 59.6–98.8) 10th year
Hsu et al. [46]	67.0 (16–97)	–	6 revisions - cage loosening - infection - reconstruction cage “failed”	–	3 broken iliac screw	6 aseptic cage loosening	3 recurrent dislocation	1 post-operative acute infection 2 deep infection	1 sciatic nerve impingement 1 vesicle-acetabular fistula 1 leg lengthening	–	6 migration > 5 mm	76% (CI 67–85) 5th year 57% (CI 39–75) 10th year
Makita et al. [50]	–	17.2 (15–18)	3 revisions in IIIA 1 revision in IIIB - aseptic loosening - dislocation - broken plate	11.1 (± 2.0)	2 broken plate 2 broken fixation screw	1 aseptic cup loosening	1 recurrent dislocation	0	3 non-union of the great trochanter (1th year osteosynthesis)	–	6 worn cups	98.2% (CI 92–100) 8th year 94.5% (CI 88–100) 10.6th year 90.7% (CI 81–100) 12.5th year 85.1% (CI 71–99) 15.2th year
Peng et al. [31]	86 (70–90)	–	11 revisions - aseptic loosening - infection	4.5 (1.5–8.5) aseptic	–	9 aseptic loosening	0	2 deep infection	0	16 radiolucent lines < 2 mm	9 migration > 3 mm	89.4% latest FU
Piriou et al. [28]	–	16.6	7 revisions - aseptic transplant resorption - infection	1.3 (± 0.9)	–	–	2 post-operative dislocation (< 3 months)	1 post-operative acute infection (< 3 months) 1 deep infection (3rd year)	–	3 radiolucent lines < 1 mm	2 cage migration > 3 mm (6th, 9th year) 5 aseptic allograft resorption (averaged at 1.2 years)	–
Piriou et al. [29]	–	Pain 5.3 Function 5.6 ROM 4.3	26 revisions - aseptic loosening - dislocation - fatigue acetabulum fracture - infection	10.4 (2.4–16.7)	–	19 aseptic loosening	9 recurrent dislocation	1 deep infection (6th year)	2 fatigue acetabulum fracture (both 0,5 year) 3 pulmonary embolism 2 femoral nerve palsy	–	63.6% with moderate or significant superior acetabulum migration	88.5% (CI 88.4–88.6) 10th year 77.8% (CI 77.7–77.9) 12th year
Prieto et al. [35]	79.0 (45–100)	–	8 revisions - aseptic loosening - dislocation - periprosthetic fracture - nerve irritation - infection	–	–	1 aseptic loosening	3 recurrent dislocation	1 peri-prosthetic infection	1 peri-prosthetic fracture 2 sciatic nerve palsy 1 femoral loosening	–	3 aseptic allograft resorption	90% 5th year 88% 10th year
Regis et al. [45]	75.6 (46–97)	–	9 revisions - aseptic loosening - infection - cage fracture	–	1 cage fracture	5 aseptic cage loosening (3–12 years)	6 post-operative dislocation	3 deep infection (early post-operative)	1 sciatic nerve palsy 1 femoral nerve palsy 2 deep vein thrombosis	–	4 migration/definite loose	80% (CI 72.6–88.1) 18.9th year
Sporer et al. [36]	–	10	5 revisions - aseptic loosening	5.3 (± 2.4)	–	5 aseptic loosening (2.6th, 3rd, 4.8th, 7.6th, 8.7th year)	1 post-operative dislocation	–	–	–	1 radiographic loose (at 2.6 year)	78% (CI 74–82) 10th year

– Information not available, *CI* confidence interval, *F* female, *FU* Follow-up, *HHS* Harris Hip Score, *M* male, *MdA* Merle d’Aubigné Score, *No.* number, *ROM* Range of Motion, *SD* standard deviation

SPSS statistical program 26.0 (SPSS, Chicago, IL, USA) and Comprehensive Meta‐Analysis software, Version 3 (Biostat, Englewood, NJ, USA) were used for data analysis. Missing mean values and variances were estimated by median, range and sample size [21]. Chi^2^ statistics was used to investigate differences in allograft type by bone loss classification. Kendall rank correlation evaluated the association between frequency of allograft use and Paprosky grade. Mann–Whitney *U* test or Kruskal–Wallis *H* test was applied to investigate outcome differences between fixation method, allograft type and reconstruction devices.

For the main outcome variable, success rate, the effect size (proportion) was calculated combined in pooled effect proportion meta-analyses. The results were depicted with 95% confidence intervals (CI) using a random-effect model analysis [22]. *I*^2^-statistic was used to quantify the degree of heterogeneity. Values of 25, 50 and 75% correlate to predefined thresholds for low, medium and high degrees of heterogeneity [23]. All *p* values are two-sided with an alpha set at 0.05.

## Results

### Literature search results

**Fig. 1:**
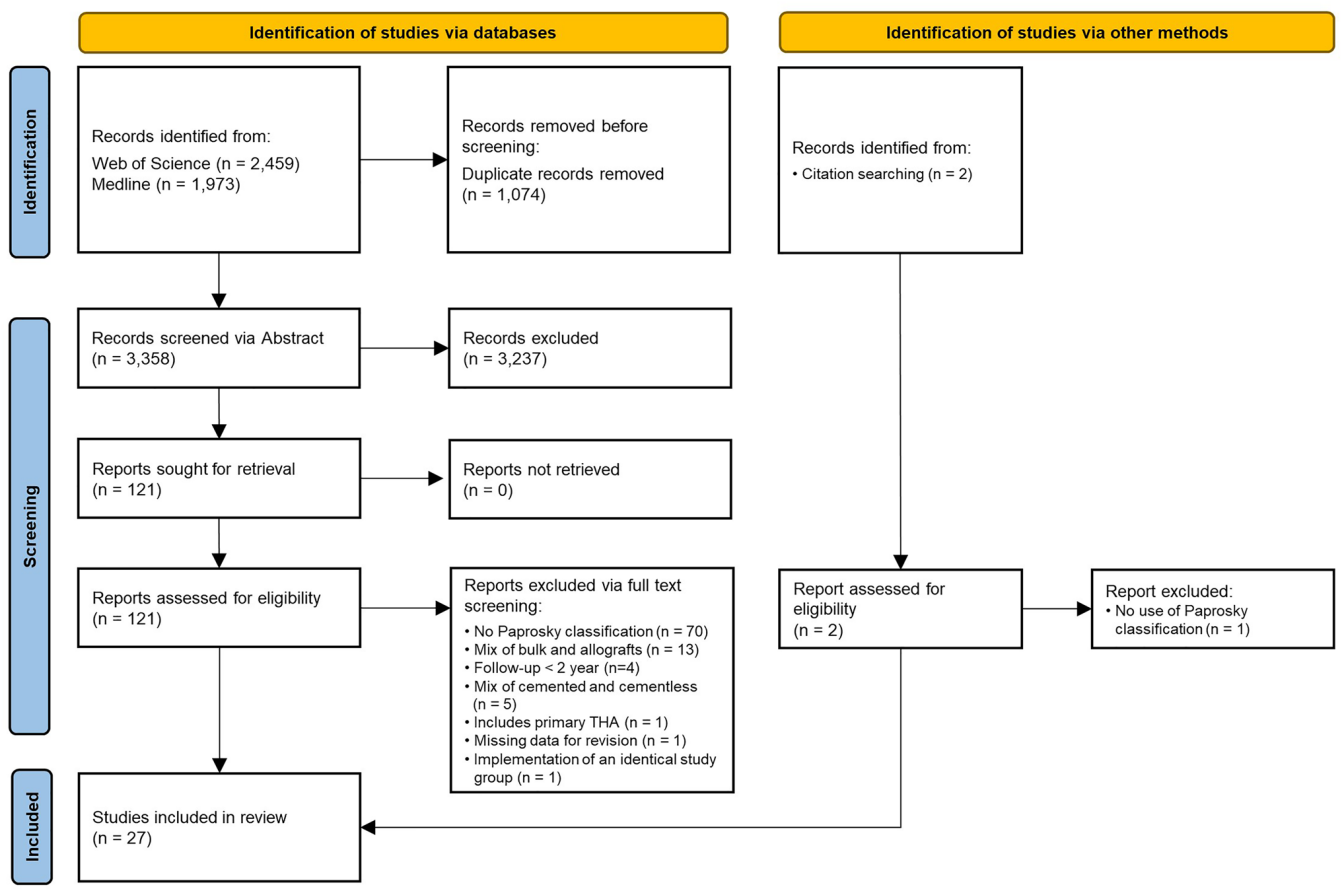
Flow chart of the systematic literature search

A total of 2459 studies obtained in Web of Science and 1973 records in Medline resulted in a list of 4432 possible studies. After excluding duplicates (*n* = 1074) and screening titles and abstracts for eligibility (*n* = 3358), 121 articles remained. Following full text review, 26 studies met the inclusion criteria and were included in this review. One additional study was added after identification through citation searching, resulting in 27 articles (Fig. 1).

### Study characteristics

All included articles are retrospective follow-up studies published between 2003 and 2021. The Black and Downs quality assessment indicated poor quality in 18 cases and fair quality in 9 cases (Suppl. Table 2). Study characteristics and outcomes are described in Table 2. In total 1561 cases of rTHA (range 20–304 per study) from 1491 patients with an average age of 63.9 years (range 22–95) were included. The average follow-up period of 7.9 years ranged from minimum 2–22 years. The main indications for rTHA were aseptic loosening (*n* = 1224), infection (*n* = 35), recurrent dislocation (*n* = 13), stem fracture (*n* = 1) or other (e.g., adverse reaction to metal debris, failed bulk allograft) (*n* = 3). Seven studies did not report the indication for revision surgery [24–30].

In total, rTHA with allograft use was performed in 31 cases with Paprosky type 1, 135 cases with type 2A, 213 cases with type 2B, 135 cases with type 2C, 502 cases with type 3A and 309 cases with type 3B defects. Three studies did not provide the specific subtype of the classification, instead only the main Paprosky category was provided [26, 31, 32]. A wide range of different reinforcement components were used for acetabular reconstruction. The most frequently used reconstruction systems were cages applied in 539 cases, followed by meshes (331 cases), cementless cups (317 cases), cemented cups (188 cases), TM cups (78 cases), TM acetabular augments with cemented cup (58 cases) and customized special cups (50 cases). In 18/27 studies cemented acetabular cup fixation was used (Table 2). Related to the pooled sample size this represents 71% all cases.

Both, the acetabular and femoral component were revised in most cases. In 405/1273 of the cases (31.8%) solely the acetabular component was revised. Eight studies did not address information about the femoral component [26, 30, 33–38]. Allografts were utilized in all cases. In 17/27 studies (63%) morselized allografts were implanted. Six studies reported on peri-prosthetic fractures caused by rTHA [35, 39–43]. These occurred in 15/1561 revisions (1%) and were treated intraoperatively. Following surgery, 17/1561 cases (1%) were diagnosed with transient or permanent nerve palsy, primarily affecting the femoral or sciatic nerve [27–30, 34, 40, 41, 43–46].

### Graft type application according to Paprosky bone defect

Four studies (14.8%) included Paprosky type 1 to 3 bone defects [29, 40, 42, 47], whereas 8 studies (29.6%) described only cases with type 2 or 3 [31–35, 38, 43, 48]. Fifteen studies (55.6%) exclusively included Paprosky type 3A and type 3B, respectively [24–28, 30, 36, 37, 39, 41, 44–46, 49, 50].

**Fig. 2:**
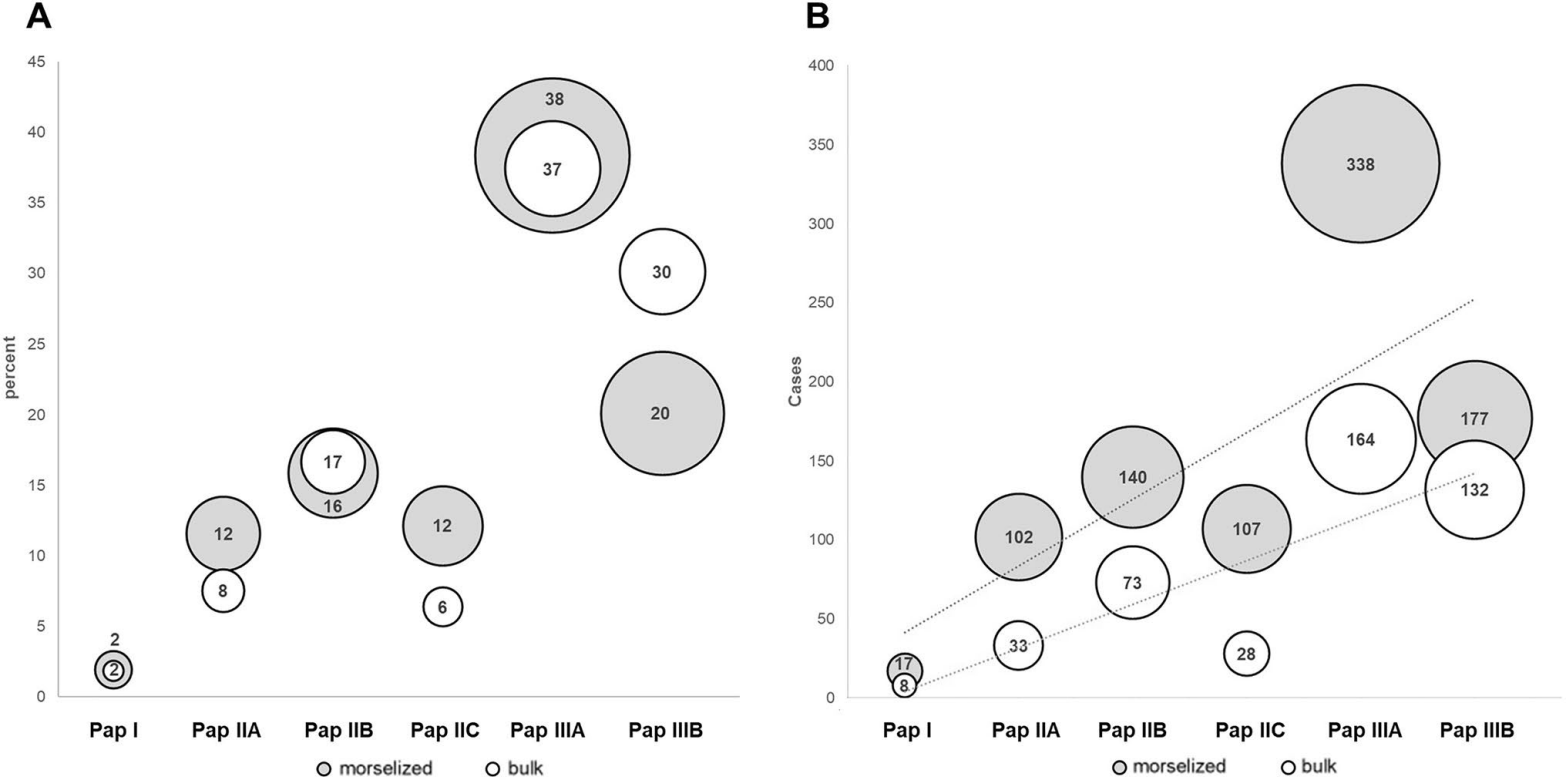
Correlation between graft use and Paprosky classification. **A** Use of morselized and bulk allografts for each Paprosky grade as percentage per total cases. **B** Total number of cases for each Paprosky grade

Considering bone grafts, morselized allografts (*n* = 998) were nominally utilized more often than bulk allografts (*n* = 563) in all type of defects (Chi^2^ = 121.1; *p* < 0.001). However, the percentage distribution suggests that the grafts are used at a similar proportion and independently of the Paprosky grade (Chi^2^ = 4.84; *p* = 0.435; Fig. 2A). Although there was a trend that bulk allografts were more likely used with higher Paprosky grades, there was no statistical difference between the use of morselized and bulk allografts in any of the defect types (*p*_Pap. 1–3_ > 0.05). Namely, in Paprosky type 2 acetabular defects, morselized grafts were implanted in 39.6% and bulk allografts in 30.6% of the cases. In type 3, morselized grafts were applied in 58.4% and bulk allografts in 67.7%. The overall use of allografts increased significantly with the type of the acetabular defect (*r*_Kendall_ = 0.69, *p* = 0.049; Fig. 2B).

### Global success rate of revision total hip arthroplasty using allografts

**Fig. 3:**
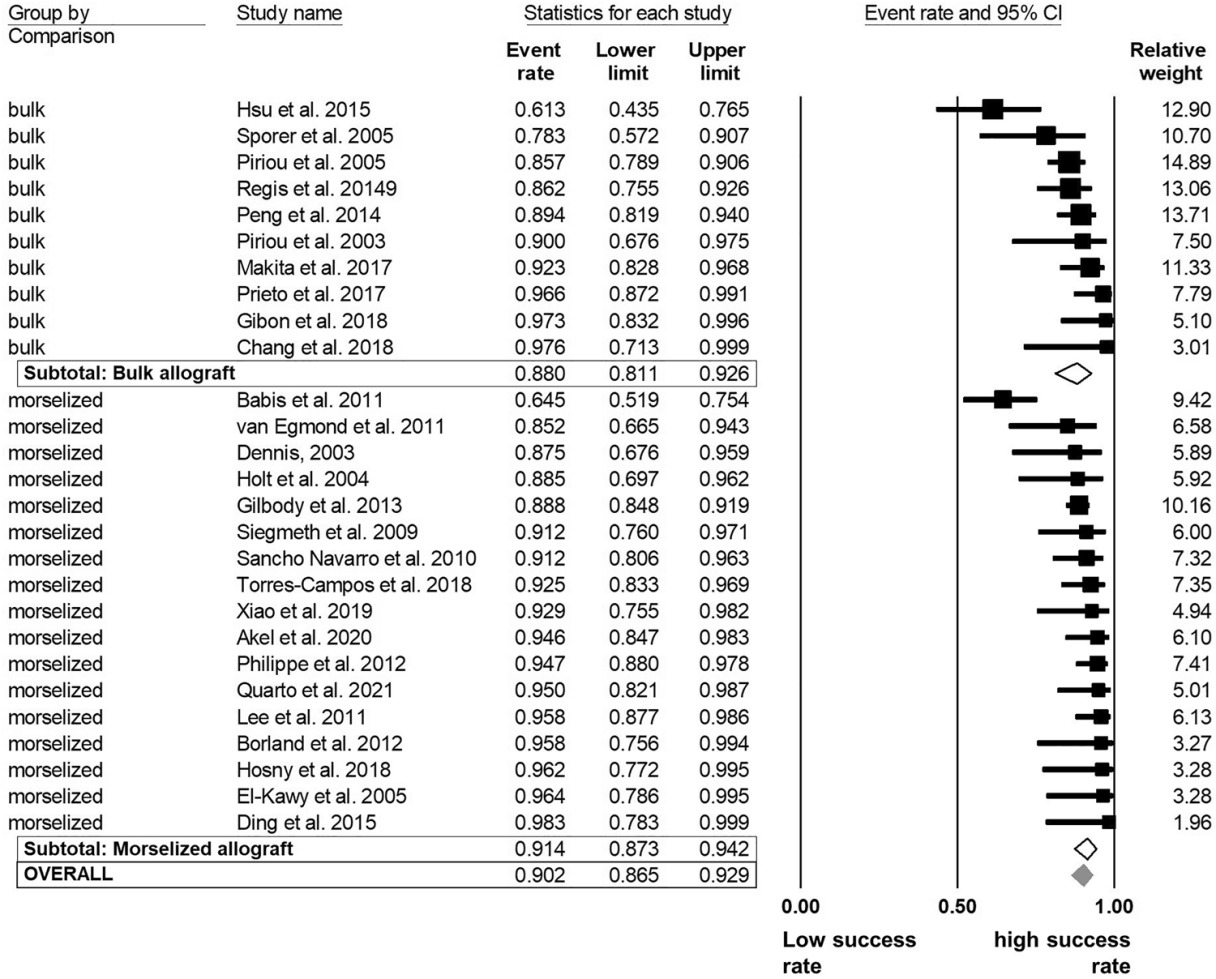
Random model meta-analysis forest plot for success rate of rTHA compared by allograft type

**Fig. 4:**
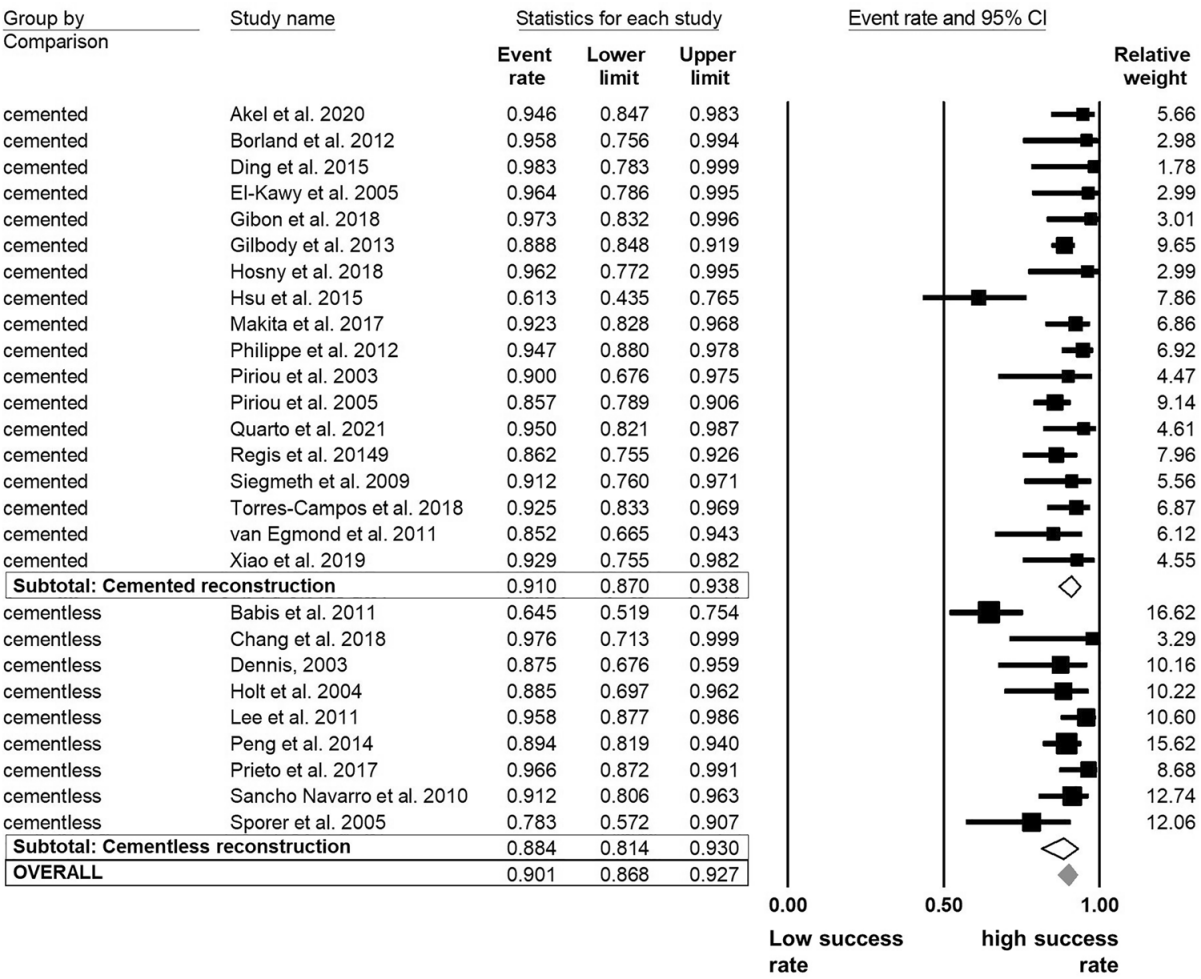
Random model meta-analysis forest plot for success rate of rTHA compared by fixation method

The overall success rate ranged from 61.3% [46] to 98.3% [34] in the examined follow-up period. The pooled estimates of success were 90% [95% CI 87–93]. The analysis demonstrated that the success rates did not differ significantly regarding to allograft type and acetabular cup fixation methods. Morselized allografts (91% [87–94]) demonstrated a 3% higher success rate than bulk allografts (88% [81–93] (Fig. 3). The *I*^2^-statitic indicated a medium degree level of data heterogeneity (*I*^2^ = 66.4%). The acetabular fixation method (cemented vs. cementless) was further evaluated to determine whether the method had an influence on the overall success rate. The analysis demonstrated no significant difference of the rTHA success between cemented (91% [87–94]) and cementless (88% [81–93]) procedures (*I*^2^ = 66.3%; Fig. 4).

**Fig. 5:**
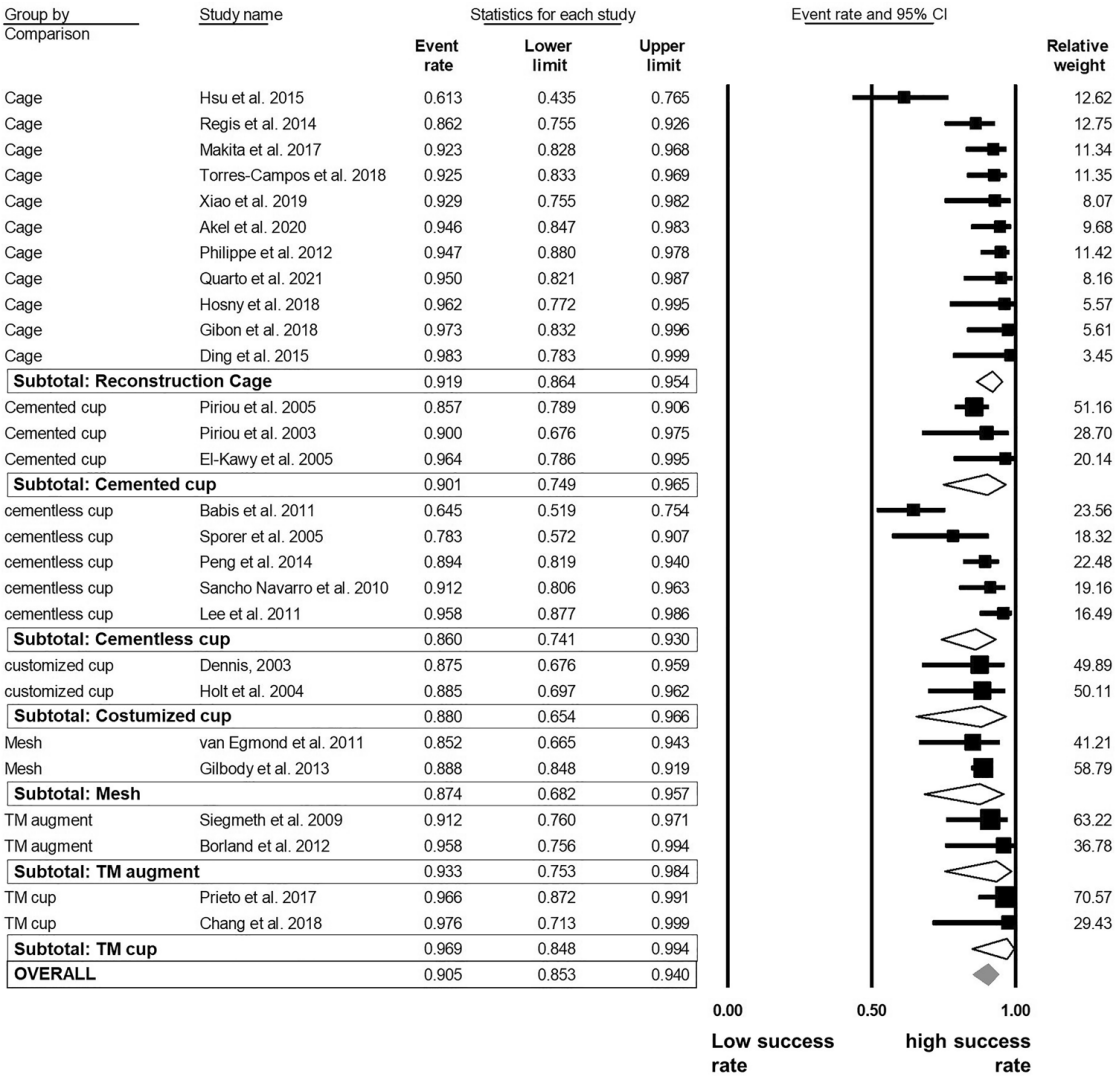
Random model meta-analysis forest plot for success rate of rTHA compared by reconstruction device and cup type

We also evaluated the influence of different cup types and reinforcement devices. No significant differences were found between the types of acetabular fixation systems in term of success rate (Fig. 5), though there was a trend for TM acetabular cups to have the highest success rate (97% [85–99]), followed by TM augments (93% [75–98]) and reconstructions cages (92% [86–95]).

### Success rate in relation to allograft type, stratified by acetabular fixation method

**Fig. 6:**
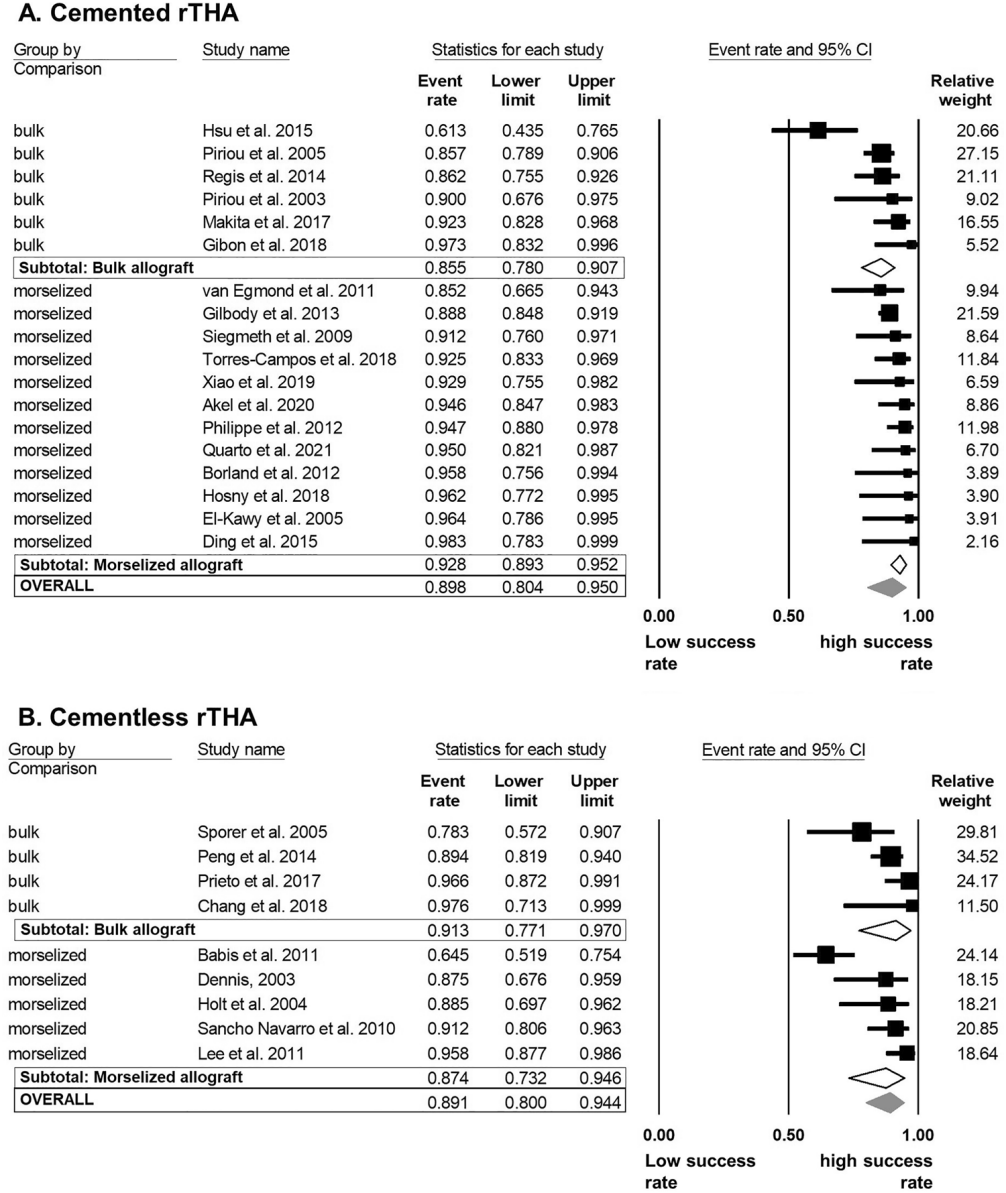
Random model meta-analysis forest plot for success rate of rTHA compared by allograft type stratified by acetabular fixation method. **A** Forest plot for allograft type comparison within the group of cemented rTHA. **B** Forest plot for allograft type comparison within the group of cementless rTHA

Since no global differences were found in the preceding meta-analyses, we further investigated allograft types and reinforcement devices within each specific fixation method. 18/27 studies (66.7%) applied a cemented cup fixation. Within the group of cemented rTHA the pooled overall estimate of success was 90% [80–95]. Level of heterogeneity was medium (*I*^2^ = 55.4%). In cemented rTHA, the success rate was 7% lower when using bulk allografts (85.5% [78–91]) than using morselized allografts (92.8% [89–95]) (Fig. 6 A). Yet, this difference was not statistically significant. In contrast, the pooled overall estimate of success within the group of cementless rTHA was 89% [80–94] with a success rate of 91% [77–97] for bulk and 87% [73–95] for morselized allografts, demonstrating a non-significant trend for clinical improved results using bulk allografts in cementless rTHA (Fig. 6B).

### Success rate in relation to reconstruction option, stratified by acetabular fixation method

**Fig. 7:**
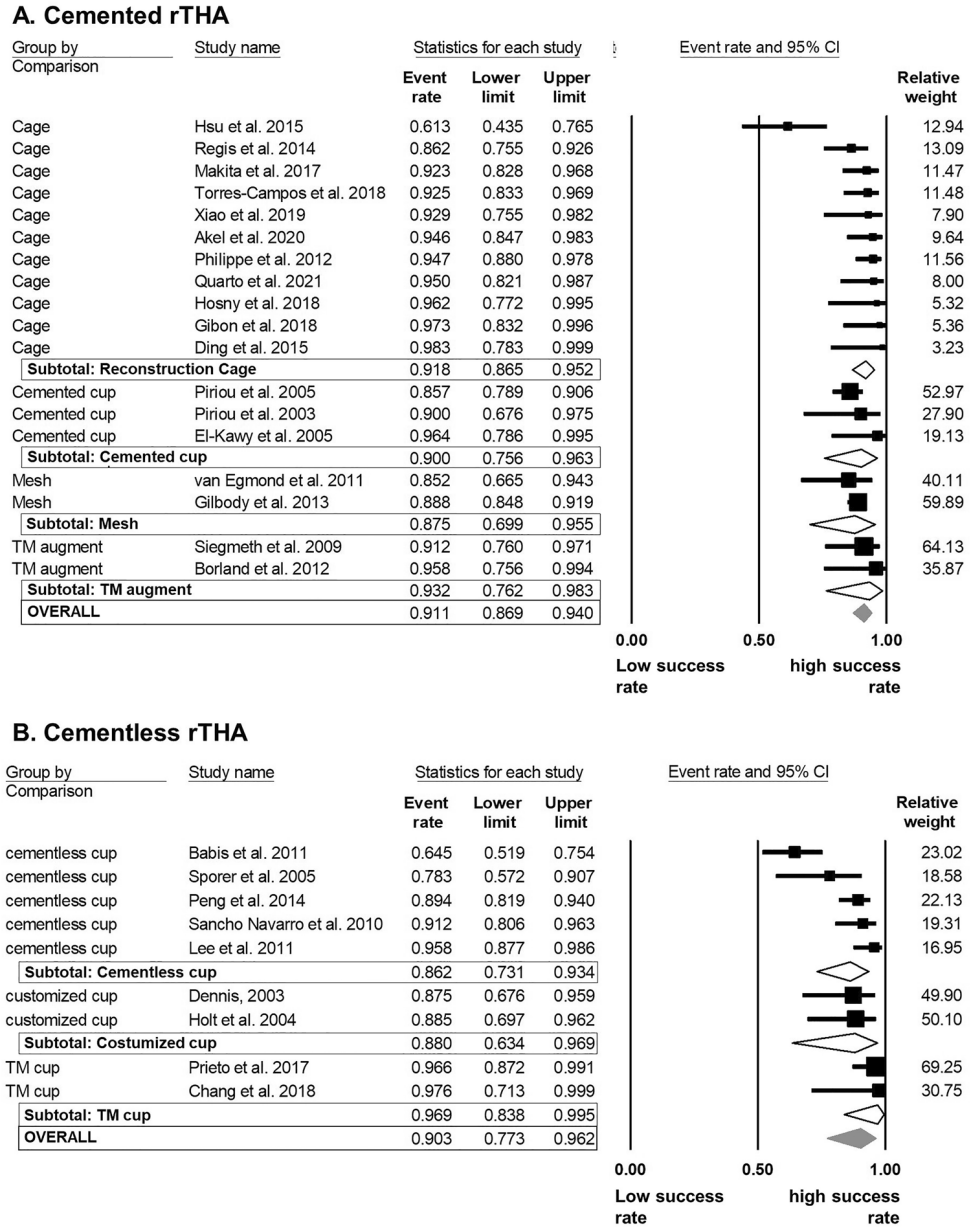
Random model meta-analysis forest plot for success rate of rTHA compared by implanted reconstruction system stratified by acetabular fixation method. **A** Forest plot for reconstruction system comparison within the group of cemented rTHA. **B** Forest plot for reconstruction system comparison within the group of cementless rTHA

In cemented rTHA, the success rates did not differ significantly between different reconstruction devices. However, TM augments provided the best (93% [76–98]) and Mesh reconstruction the worst (87% [70–96]) success within this subgroup (Fig. 7A). In the group of cementless rTHA, no significant differences between cementless cups (86% [73–93]), customized cups (88% [63–97]) and TM cups (97% [84–100]) were detected (Fig. 7B). Nonetheless, TM cups had a success rate approximately 10% higher than the other comparative reconstruction devices.

### Improvement of clinical outcomes

In addition to success rates, the clinical outcomes of the included studies were analyzed. The Harris hip score (HHS) was applied in 16 studies [24, 26, 27, 30–35, 37, 43–47, 49] and the modified Merle d’Aubigne (MdA) score in 9 studies [28, 29, 32, 36, 39–42, 50]. All publications described improved post-operative outcome scores. At last follow-up, the average HHS was 74.6 (range 15–100) points indicating ‘fair’ results [51] and the average MdA measured 15.2 (range 7–18) points representing ‘good’ clinical results[52] at last follow-up. The pooled mean improvement in HHS was 38.5 (range 16.3–54.4) points and the mean MdA improvement amounts 5.9 (range 4.0–9.9) points. The outcome improved independently of the use of bone cement (*U* test = 23; *p* = 0.448), allograft type (*U* test = 20; *p* = 0.396) and reconstruction device (*H* test = 6.415, *p* = 0.268).

## Discussion

Revision total hip arthroplasty is a complex surgical procedure, which can be particularly challenging in situations of severe acetabular bone loss. Bone grafts and various reconstruction systems are widely used during rTHA to address severe bone loss. The purpose of this systematic review and meta-analysis was to evaluate the mid- and long-term outcome of different reconstruction options using allografts in rTHA. Overall, no differences between allograft types, fixation methods, reconstruction systems, and cup types were observed. Notably, all included studies were retrospective follow-up studies without control groups (evidence level: Type IV). Therefore, no high-level evidence on the use of allografts with different reconstruction devices could be reported, which is also reflected by the mainly poor quality in the Downs and Black quality assessment.

This meta-analysis presents the pooled success rates at an average follow-up period of 7.9 years. Findings from 27 studies demonstrated a pooled success rate of 90% [0.80–0.95], accounting for implant failures, aseptic loosening, and infections. The most reported complication was aseptic loosening (6% [4–9]), followed by dislocation (5% [4–7]), and in equal proportions implant failure (3% [2–5]) and infections (3% [2–4]). Aseptic loosening was recorded in 20/27 publications. Twelve of 17 studies reported aseptic loosening in an average of 6.8% of the cases with morselized grafts [27, 30, 32, 33, 37, 38, 40, 41, 43, 44, 47, 49] and in 8/10 studies (80.0%) in 8.3% of the cases with bulk allografts [29, 31, 35, 36, 39, 45, 46, 50]. While the aseptic loosening rate was 6.6% in cemented rTHA, cementless procedures reached a loosening rate of 9.2%. Since radiographic radiolucent lines and cup migration could not be included in the success variable in standardized manner, it is likely that the aseptic loosening rate is probably fairly higher.

A postulated, clinically important predictor for the outcome is the reconstruction method [53]. Reconstruction rings with cemented cups are considered as reliable reconstruction method. Current systematic reviews and meta-analyses have previously studied the options for managing severe acetabular bone loss [1, 14, 15]. Trabecular metal augments and shells may demonstrate the lowest revision and radiographic loosening rate while bone grafting with metal mesh has not been recommended for the reconstruction of pelvic discontinuity and type 3B bone loss [14]. Complementary to these previous findings, this review included four studies reporting results on TM reconstruction devices, two of these using impacted morselized grafts with augments and cemented cups in 58 cases [25, 38]. The other two studies described the results of 78 cases treated with cementless TM cups [26, 35]. TM systems achieved the highest success rates in this meta-analysis, which is why these devices may represent a promising option to address severe acetabular bone defects. In total, studies with TM reconstruction systems report a pooled loosening rate of 2.2%, a dislocation rate of 3.7%, and an implant failure rate of 1.4%. Still, the number of included studies is small, which may be the reason for the lack of significant differences in comparison with other acetabular reconstruction devices.

Regarding the type of allograft, 17 studies used exclusively morselized and ten studies bulk allografts. Studies evaluating bulk allografts reported that gaps between graft and host bone were filled with additional morselized grafts. The long-term results of morselized and bulk allografts did not differ significantly. Morselized allografts, however, had a 2% higher overall success rate. When stratified by fixation, a trend towards improved outcome with cementless fixation and poorer outcome with cemented fixation was observed for bulk compared to morselized allografts. In search of mechanisms for aseptic loosening, we have previously demonstrated histological evidence of more fibrous ingrowth in cases with morselized compared to bulk allografts during rTHA with cemented cup fixation [12, 13]. Although this appears somewhat contrary to the results of the present meta-analysis, the extent to which fibrous ingrowth at the cement-allograft or allograft-host bone interface contributes to aseptic loosening remains unknown. It is also likely that other factors (e.g., surgical technique, host-related factors) also influence the outcome.

In most articles, complications were presented in an aggregated format across all bone defect types. Only 2/27 studies categorized complications by Paprosky bone loss type [30, 40]. Consequently, revisions and complications that have occurred cannot be related to a specific type of bone defect. It can be suggested that the lower success rate for bulk allografts is attributed to the preference for applying these types of grafts in more severe acetabular defects. However, since both graft types were applied in equal proportion in all Paprosky classifications, it may be concluded that bulk allografts tend do have a higher complication rate than morselized allografts independent of the applied acetabular reconstruction system.

On average, the clinical outcome of the examined patients, measured with HHS and MdA score, was good. Both scores are established and well validated instruments for the objective assessment of the functionality of the hip and reflect the subjective pain intensity. Overall, the results described good and encouraging mid- to long-term results of acetabular reconstruction with both cages and TM augments or shells using bulk or morselized allografts, supporting their use for severe bone loss. There were no significant differences between the reconstruction systems, fixation methods or graft types but descriptive slightly improved outcomes for the use of TM systems and morselized allografts. The method of acetabular cup fixation solely had no influence on the success or revision rate. Also, no significant differences were observed between cemented and non-cemented cups.

### Strength and limitations

This study has clear strengths. While previous systematic reviews on various reconstruction options for acetabular defects were heterogeneous and not comparable, this review is focusing on different reconstruction options that included allograft use with precise inclusion and exclusion criteria. Our search strategy allows the meta-analytical comparison of different influence factors affecting the outcome of rTHA, e.g., reconstruction device, fixation method, graft type.

The main limitation of this systematic review is that the source studies did not provide high-quality evidence. In view of fact that, contrary to Paprosky's original recommendation [5], morselized allografts were also used for type 3A and 3B bone defects, it should be considered to compare the use of bulk and morselized allografts in a randomized controlled trial to eliminate confounding variables. The differences in surgical approach, postoperative care protocols, age at primary revision, patient demographics, primary disease, number of cases (range 20–304), and time to follow-up (range 2–22 years) created limited comparability. However, the calculated *I*^2^-statistics for heterogeneity was medium in any of the meta-analyses conducted.

Two of the largest scientific databases for medical literature were systematically searched. When identifying studies, there was the difficulty that all cases had to be treated with an allograft. Often allografts were not primary outcome, resulting in the exclusion of numerous studies that did not clearly demonstrate that all patients received a bone graft. However, this approach has concurrently increased the consistent character of the studies. Furthermore, metanalytical results were affected by publication bias [54, 55], meaning the tendency not to publish studies with non-significant or unfavorable results. Overall, the quality of the included study is low. No randomized-controlled trials were found to be included in the meta-analysis. However, for the outcome parameters examined, e.g., complication rate, only follow-up studies were suitable and applicable.

## Conclusion

To our knowledge, this is the first systematic review analyzing the outcome of rTHA with allograft use in acetabular defects. TM system and cages appear to have the best success rate. The results further suggest that morselized and bulk allografts are used in equal proportions in all Paprosky grades, with no significant differences in clinical outcome. Since only retrospective studies could be included, additional research is needed to finally address this question.

## Supplementary Information

Below is the link to the electronic supplementary material.

Supplementary file1 (DOCX 29 KB)

## Data Availability

All data generated or analysed during this systematic review and meta-analysis are included in this published article, tables and its supplementary documents.
