# Supplementary material for: Outcome of different reconstruction options using allografts in revision total hip arthroplasty for severe acetabular bone loss: a systematic review and meta-analysis
Source: Arch Orthop Trauma Surg. 2023 Mar 27;143(10):6403–22. doi: 10.1007/s00402-023-04843-9 (PMC10491513; doi:10.1007/s00402-023-04843-9)
Supplement: Supplementary file 1 — Supplementary file1 (DOCX 29 KB) [file 402_2023_4843_MOESM1_ESM.docx]

**Suppl. Table 1: Literature search history**

| **Database** | **Search terms** |
| --- | --- |
| Medline via OVID | ((Acetabulum.mp. or Acetabulum/) OR Acetabular.mp. OR "Bone defect*".mp. OR Bone def*.mp. OR Bone loss.mp.) **AND** (Allografts/ OR (Allograft.mp. or Allografts/) OR Composite graft.mp. OR (transplants/ or transplant.mp) OR graft.mp. OR cage.mp. OR Bone Transplantation/ OR "Bone graft".mp. OR Ring.mp.) **AND** (Arthroplasty, Replacement, Hip/ or Hip Prosthesis/) OR (Reoperation/ or Reoperation.mp) OR Arthroplasty, Replacement, Hip/ OR Revision.mp. or Hip Prosthesis/ or Arthroplasty, Replacement, Hip/) **NOT** (exp rat/ or rat*.mp. or exp mouse/ or mice.mp. or Mice/) **AND** limit yr="1990-2021") |
| Web of Science | ((TS=Acetabulum OR ALL=Acetabulum) OR (TS=Acetabular OR ALL=Acetabular) OR TS="Bone defect*" OR ALL="Bone defect*" OR ALL="Bone def*" OR TS="Bone def*" OR (TS=Bone loss OR ALL=Bone loss)) **AND** ((TS=Allograft OR ALL=Allograft) OR (TS=Composite graft OR ALL=Composite graft) OR TS=graft OR ALL=graft OR TS=cage OR ALL=cage OR (TS=Bone Transplant* OR ALL=Bone Transplant*) OR (TS="Bone graft" OR ALL="Bone graft") OR ALL=Rings) **AND** ((TS=Reoperation OR ALL=Reoperation) OR (TS=Revision OR ALL=Revision) OR (TS= Hip Arthroplasty OR ALL=Hip Arthroplasty OR TS= Hip replacement OR ALL=Hip replacement OR TS=Hip Prosthesis OR ALL=Hip Prosthesis)) **NOT** TS=(rat OR rat* OR mouse OR mice) **AND** limit yr="1990-2021") |

**Suppl. Table 2: Modified Downs and Black checklist for evaluating the methodological quality of included studies (n = 27)**

|  | **Reporting** | | | | | | | | | | **External Validity** | | | **Internal Validity (Bias)** | | | | | | | **Internal Validity (Confounding)** | | | | | | **P** | **D&B Score** |
| --- | --- | --- | --- | --- | --- | --- | --- | --- | --- | --- | --- | --- | --- | --- | --- | --- | --- | --- | --- | --- | --- | --- | --- | --- | --- | --- | --- | --- |
| **Study** | **1** | **2** | **3** | **4** | **5*** | **6** | **7** | **8** | **9** | **10** | **11** | **12** | **13** | **14** | **15** | **16** | **17** | **18** | **19** | **20** | **21** | **22** | **23** | **24** | **25** | **26** | **27** | **∑** |
| Akel et al., *2019* | Y | Y | Y | N | N | Y | Y | Y | N | Y | Y | U | Y | N | N | Y | Y | Y | Y | Y | N | N | N | N | N | N | N | 14 |
| Babis et al., *2011* | Y | Y | Y | Y | N | Y | Y | Y | U | U | Y | U | Y | N | N | Y | Y | Y | Y | Y | N | N | N | N | N | U | N | 14 |
| Borland  et al., *2021* | Y | Y | Y | Y | N | Y | Y | Y | U | Y | Y | U | Y | N | N | Y | Y | Y | Y | Y | N | N | N | N | N | U | N | 15 |
| Chang et al., *2018* | Y | Y | Y | Y | N | Y | Y | Y | N | N | Y | U | Y | N | N | Y | Y | Y | Y | Y | N | N | N | N | N | N | N | 14 |
| Dennis,  *2003* | N | N | Y | Y | N | Y | Y | Y | U | U | Y | U | Y | N | N | U | N | N | Y | Y | N | N | N | N | N | U | N | 9 |
| Ding et al., *2015* | Y | Y | Y | N | P | Y | Y | Y | N | N | Y | U | Y | N | N | Y | N | Y | Y | Y | N | N | N | N | N | N | N | 13 |
| El-Kawy  et al., *2005* | Y | N | Y | Y | N | Y | Y | Y | U | U | Y | U | Y | N | N | U | U | N | Y | Y | N | N | N | N | N | U | N | 10 |
| Gibon et al., *2018* | Y | Y | Y | Y | N | Y | Y | Y | N | Y | Y | U | Y | N | N | Y | Y | Y | Y | Y | N | N | N | N | N | N | N | 15 |
| Gilbody  et al., *2013* | Y | Y | Y | Y | P | Y | Y | Y | U | Y | Y | U | Y | N | N | Y | Y | Y | Y | Y | N | N | N | N | Y | U | N | 16 |
| Holt et al., *2004* | Y | Y | Y | Y | N | Y | Y | Y | U | U | Y | U | Y | N | N | U | N | N | Y | Y | N | N | N | N | N | U | N | 11 |
| Hosny et al., *2008* | Y | Y | Y | Y | N | Y | Y | Y | U | N | Y | U | Y | N | N | N | Y | Y | Y | Y | N | N | N | N | N | U | N | 13 |
| Hsu et al., *2015* | Y | Y | Y | Y | N | Y | Y | Y | N | Y | Y | U | Y | N | N | N | Y | Y | Y | Y | N | N | N | N | N | N | N | 14 |
| Lee et al., *2011* | Y | Y | N | Y | N | Y | N | Y | N | N | Y | U | Y | N | N | Y | Y | N | Y | Y | N | N | N | N | N | N | N | 11 |
| Makita et al., *2017* | Y | Y | Y | Y | Y | Y | Y | Y | N | N | Y | U | Y | N | N | Y | Y | Y | Y | Y | N | N | N | N | Y | Y | N | 17 |
| Peng et al., 2014 | Y | Y | Y | Y | N | Y | Y | Y | U | N | Y | U | Y | N | N | Y | Y | Y | Y | Y | N | N | N | N | N | U | N | 14 |
|  | **Reporting** | | | | | | | | | | **External Validity** | | | **Internal Validity (Bias)** | | | | | | | **Internal Validity (Confounding)** | | | | | | **P** | **D&B Score** |
| **Study** | **1** | **2** | **3** | **4** | **5*** | **6** | **7** | **8** | **9** | **10** | **11** | **12** | **13** | **14** | **15** | **16** | **17** | **18** | **19** | **20** | **21** | **22** | **23** | **24** | **25** | **26** | **27** | **∑** |
| Philippe et al., 2012 | Y | Y | Y | Y | P | Y | Y | Y | N | N | Y | U | Y | N | N | Y | Y | Y | Y | Y | N | N | N | N | Y | N | N | 15 |
| Piriou et al., 2003 | Y | Y | Y | Y | N | Y | Y | Y | U | U | Y | U | Y | N | N | Y | N | N | Y | Y | N | N | N | N | N | U | N | 12 |
| Piriou et al., 2005 | Y | Y | N | Y | P | Y | Y | Y | N | U | Y | U | Y | N | N | Y | Y | N | Y | Y | N | N | N | N | Y | N | N | 14 |
| Prieto et al., 2017 | Y | Y | Y | Y | P | Y | Y | Y | U | N | Y | U | Y | N | N | Y | Y | N | Y | Y | N | N | N | N | N | U | N | 14 |
| Quarto et al, 2021 | Y | Y | Y | Y | P | Y | Y | Y | N | Y | Y | U | Y | N | N | Y | N | Y | Y | Y | N | N | N | N | Y | N | N | 16 |
| Regis et al., 2014 | Y | Y | Y | Y | N | Y | Y | Y | U | Y | Y | U | Y | N | N | Y | Y | Y | Y | Y | N | N | N | N | N | U | N | 15 |
| Sancho Navarro  et al., 2010 | Y | Y | Y | Y | N | Y | N | Y | U | U | Y | U | Y | N | N | Y | N | N | Y | Y | N | N | N | N | N | U | N | 11 |
| Siegmeth et al., 2009 | Y | Y | Y | Y | N | Y | Y | Y | U | N | Y | U | Y | N | N | Y | N | N | Y | Y | N | N | N | N | N | Y | N | 13 |
| Sporer et al., 2005 | Y | Y | Y | Y | N | Y | N | Y | N | U | Y | U | Y | N | N | Y | N | N | Y | Y | N | N | N | N | N | N | N | 11 |
| Torres-Campos  et al., 2018 | Y | Y | Y | Y | P | Y | Y | Y | U | Y | Y | U | Y | N | N | Y | N | Y | Y | Y | N | N | N | N | Y | N | N | 16 |
| van Egmond et al., 2011 | Y | Y | Y | Y | N | Y | Y | Y | U | U | Y | U | Y | N | N | Y | Y | Y | Y | Y | N | N | N | N | N | U | N | 14 |
| Xiao et al., 2019 | Y | Y | Y | Y | N | Y | Y | Y | U | Y | Y | U | Y | N | N | Y | Y | Y | Y | Y | N | N | N | N | N | U | N | 15 |

Criteria based on modified Downs and Black checklist with a maximum of 28 points: Y (yes) = criterion met (1 point); N (no) = criterion not met (0 points); P (partial) = criterion partially met; U (unable to determine) = criterion was unable to determine (0 points); ∑ = Sum; Downs and Black score ranges corresponds to quality levels: excellent (26-28), good (20-25), fair (15-19), poor (≤14)
P Power; * category “Y” (yes) weighs 2 points and P (partial) weights 1 point in item 5
